# Temperature- and chemical-induced neurotoxicity in zebrafish

**DOI:** 10.3389/fphys.2023.1276941

**Published:** 2023-10-03

**Authors:** Mattia Toni, Chiara Arena, Carla Cioni, Gabriella Tedeschi

**Affiliations:** ^1^ Department of Biology and Biotechnologies “Charles Darwin”, Sapienza University, Rome, Italy; ^2^ Department of Veterinary Medicine and Animal Science (DIVAS), Università Degli Studi di Milano, Milano, Italy; ^3^ CRC “Innovation for Well-Being and Environment” (I-WE), Università Degli Studi di Milano, Milano, Italy

**Keywords:** neurotoxicity, temperature, zebrafish, proteomic, behaviour

## Abstract

Throughout their lives, humans encounter a plethora of substances capable of inducing neurotoxic effects, including drugs, heavy metals and pesticides. Neurotoxicity manifests when exposure to these chemicals disrupts the normal functioning of the nervous system, and some neurotoxic agents have been linked to neurodegenerative pathologies such as Parkinson’s and Alzheimer’s disease. The growing concern surrounding the neurotoxic impacts of both naturally occurring and man-made toxic substances necessitates the identification of animal models for rapid testing across a wide spectrum of substances and concentrations, and the utilization of tools capable of detecting nervous system alterations spanning from the molecular level up to the behavioural one. Zebrafish (*Danio rerio*) is gaining prominence in the field of neuroscience due to its versatility. The possibility of analysing all developmental stages (embryo, larva and adult), applying the most common “omics” approaches (transcriptomics, proteomics, lipidomics, etc.) and conducting a wide range of behavioural tests makes zebrafish an excellent model for neurotoxicity studies. This review delves into the main experimental approaches adopted and the main markers analysed in neurotoxicity studies in zebrafish, showing that neurotoxic phenomena can be triggered not only by exposure to chemical substances but also by fluctuations in temperature. The findings presented here serve as a valuable resource for the study of neurotoxicity in zebrafish and define new scenarios in ecotoxicology suggesting that alterations in temperature can synergistically compound the neurotoxic effects of chemical substances, intensifying their detrimental impact on fish populations.

## 1 Introduction

Human activities, along with natural events such as eruptions, earthquakes and floods, significantly contribute to the release of pollutants into the natural environment. Since these pollutants can have toxic effects (Deng et al., 2019), their accumulation in water and soil, as well as their entry into the food chain, poses substantial risks to various living organisms including humans.

Neurotoxicity is defined as “any adverse effect on the chemistry, structure or function of the nervous system, during development or at maturity, induced by chemical or physical influences” (Ladefoged et al., 1995; Costa, 2017). According to this definition, neurotoxicity can result not only from chemical substances but also from alterations in environmental chemical and physical parameters.

Neurotoxicity is a multifaceted field of study, as neurotoxic agents can operate at various levels, including molecular, cellular, tissue, systemic, organismal and behavioural. Furthermore, the susceptibility of nervous system to toxic insults varies depending on the developmental stage, with embryonic stages often proving more sensitive than adults. Additionally, the extent of neurotoxicity depends on the substance’s concentration and the duration of exposure, whether acute or chronic (Costa et al., 2004; Giordano and Costa, 2012; Costa, 2017).

Given the potentially vast array of substances and conditions capable of inducing neurotoxicity, the use of robust animal models is essential for testing a wide range of treatments across different developmental stages. Among vertebrates, zebrafish is gaining recognition as an excellent model for studying neurotoxicity, owing to its ecological relevance since the pollutants flow and concentrate in watercourses and water basins altering their chemical-physical parameters. Zebrafish’s methodological versatility is also a significant advantage, facilitating various experimental approaches, including “omics” (proteomics, transcriptomics, lipidomics, etc.) and behavioural approaches (Lai et al., 2021; Ivantsova et al., 2023).

Zebrafish, a small freshwater cyprinid native to the Ganges and Brahmaputra river basins in southern Asia (Parichy, 2015), can be easily and cost-effectively maintained in aquaria. Its high fecundity and rapid embryonic development allows the generation of large numbers of individuals. Initially employed primarily in developmental biology, zebrafish is now become a valuable model in various biomedical research fields, including neuroscience and neurotoxicity. The transparency of zebrafish embryos and the rapid development of their nervous system within 3 days post fertilization (Kalueff et al., 2016) make them ideal for developmental toxicity assays (Xia et al., 2018) and toxicity screenings (Lessman, 2011). Additionally, the use of transgenic lines with fluorescence-labeled tissues enables the study of tissue-specific drug effects (Sager et al., 2010). With nearly 70% DNA sequence homology to humans (Howe et al., 2013; Babin et al., 2014; Faria et al., 2015; Horzmann and Freeman, 2016), zebrafish allows for translational research. It is also a valuable resource in behavioural neuroscience (Gerlai, 2023), with numerous behavioural tests that can be applied depending on the developmental stage. Some of these tests for adult zebrafish include the novel tank diving, light and dark preference, social preference, mirror biting, and Y-Maze, validated for studying anxiety-like and boldness behaviour, social preference, aggressiveness, and explorative behaviour (Toni et al., 2019a; Angiulli et al., 2020; Fasano et al., 2021; Nonnis et al., 2021; Maffioli et al., 2022).

Zebrafish offers the flexibility to study different developmental stages, and scientific work has been conducted on embryos, larvae, juveniles or adults. A quick search on NCBI Pubmed using the search criteria “[zebrafish (Title)] AND [larva* (Title)]” or “[zebrafish (Title)] AND [adult (Title)]” revealed a higher number of studies conducted on larvae compared to adults (2,135 *versus* 1,544, as of 04/09/2023). This difference likely arises from the lower cost and time required to obtain larvae compared to adults, and possibly from current regulations. For instance, the Directive 2010/63/EU that in European countries “establishes measures for the protection of animals used for scientific purposes” and applies to live non-human vertebrate animals, including independently feeding larval forms (Toni et al., 2019b). This bureaucratic process makes experimentation on adult zebrafish, to which the legislation refers, more demanding than on larvae. In some cases, both larvae and adults can be analysed using the same experimental approaches, such as the analysis of gene and protein expression and enzymatic activity. However, in other cases, different experimental settings and tools are necessary, particularly for behavioural studies.

**FIGURE 1:**
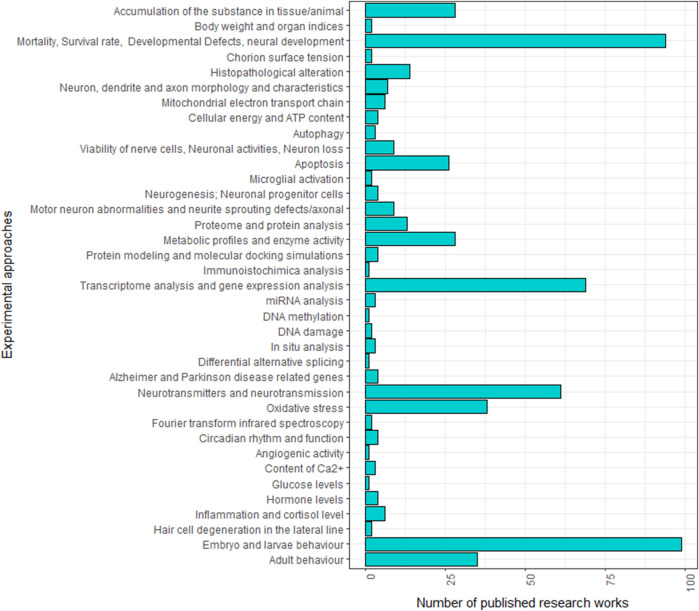
Frequencies of experimental approaches employed in zebrafish studies analysing the neurotoxic effect of specific treatments in the literature.

Over the past two decades, numerous studies have explored the neurotoxic effect of specific chemicals using zebrafish. Given the complexity of detecting nervous system alterations, a multidisciplinary approach is typically employed to investigate the neurotoxic effects across various levels, from the molecular to the behavioural. As shown in Figure 1; Supplementary Table S1, these studies do not consistently follow the same experimental protocols, making it challenging to compare the effects of different neurotoxic agents. Variations are in fact observed in neurotoxicity markers and developmental stages (embryo, larva or adult) analysed across studies.

**FIGURE 2:**
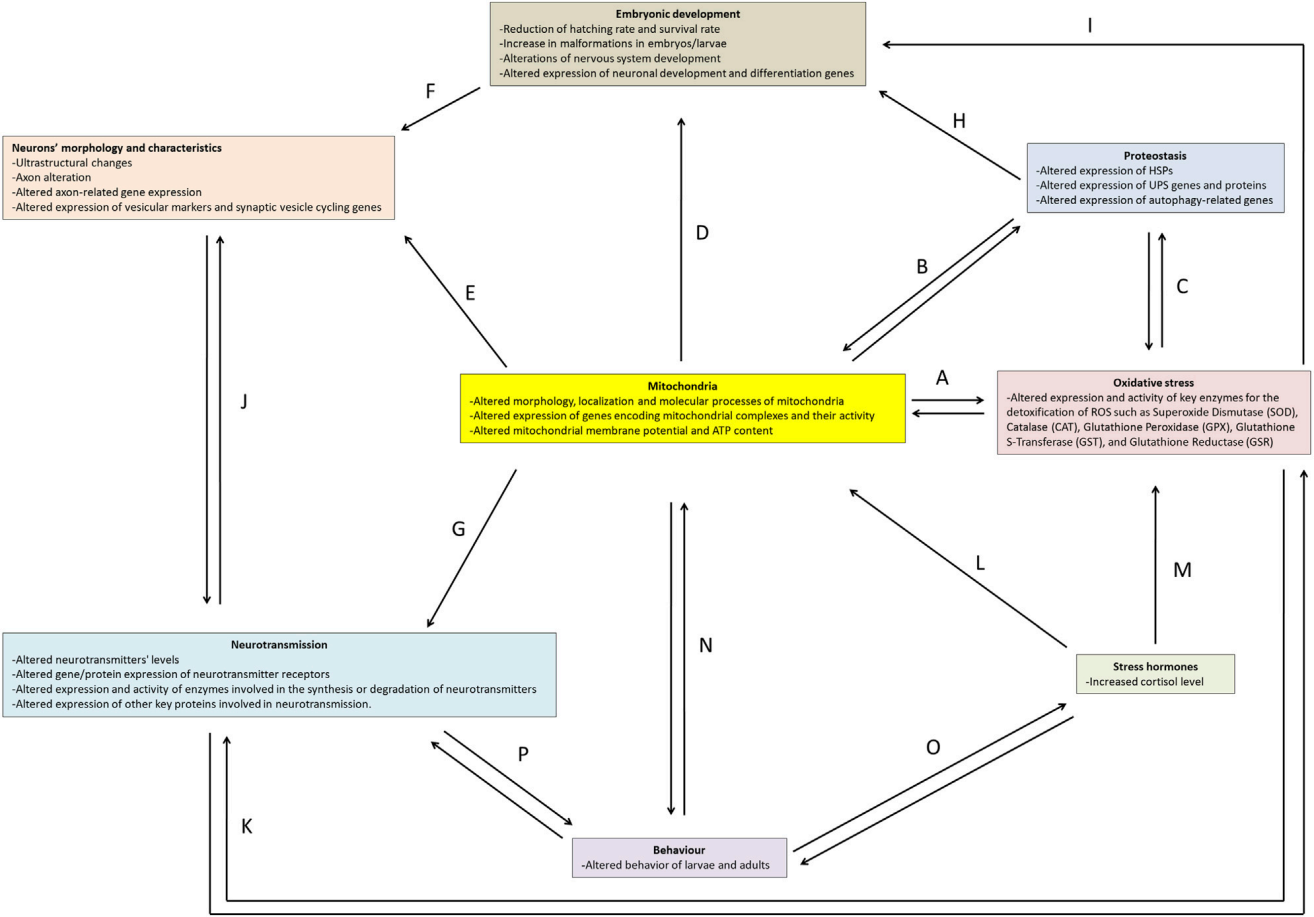
Main markers and effects of neurotoxicity in zebrafish. The arrows **(A-P)** indicate the correlation network between the topics.

Experimental evidence demonstrates that numerous chemicals and thermal fluctuations can induce neurotoxic effects in zebrafish. This work aims to provide an overview of key experimental methodologies, the markers utilised in neurotoxicity investigations, and the effects of both thermal and chemical treatments in zebrafish. Specifically, we will address aspects of embryonic development, proteostasis, stress hormones, oxidative stress, mitochondria, neuron morphology, neurotransmission, and behaviour (Figure 2).

## 2 Neurotoxic effect on embryonic development

The first toxic effect that is usually considered when testing a chemical treatment in zebrafish is its impact on fish development. The hatching rate (Jin et al., 2019a; He et al., 2020; Baran et al., 2021; Guo et al., 2021; Park et al., 2021; Oh et al., 2022), survival rate (Yao et al., 2017; Liu et al., 2019; Park et al., 2020; Wu et al., 2021; Baran et al., 2021; Oh et al., 2022), and malformations in embryos and larvae can be considered (Figures 1, 2; Supplementary Table S1). For instance, reduction in hatching rate has been observed following exposure to substances as acrylamide (Park et al., 2021), lead (Jin et al., 2019a), propofol (He et al., 2020), olaquindox (Guo et al., 2021), butylated hydroxyanisole (BHA) and tertiary butylhydroquinone (TBHQ) (Baran et al., 2021). Likewise, decreased survival rates have been reported after treatment with 1-methyl-4-phenyl-1,2,3,6 tetrahydropyridine (Yao et al., 2017), fipronil (Wu et al., 2021), aflatoxin B1 (Park et al., 2020), lead (Liu et al., 2019), BHA and TBHQ (Baran et al., 2021). It's worth noting that variations in temperature can significantly impact embryonic development, with optimal survival occurring within the 22°C–32°C range but decreasing to approximately 43% at 34°C, while being negligible below 22°C and above 34°C (Schnurr et al., 2014). Additionally, the embryonic development rate increases between 22°C and 34°C but decreases beyond temperature threshold (Schnurr et al., 2014).

Noteworthy malformations identified in toxicity studies involving chemically treated zebrafish include pericardial and heart edema (Ren et al., 2018; Li et al., 2019; Liu et al., 2019; Park et al., 2019; He et al., 2020; Baran et al., 2021; Muhsen et al., 2021; Park et al., 2021), yolk alteration (Guo et al., 2015; Yao et al., 2017; Ren et al., 2018; Jin et al., 2019a; Liu et al., 2019; Baran et al., 2021; Muhsen et al., 2021; Park et al., 2021; Wang et al., 2021), curved body axis and scoliosis (Jin et al., 2019a; Park et al., 2019; Li et al., 2020; Baran et al., 2021; Park et al., 2021), and swim bladder deficiency (Jin et al., 2019a; Park et al., 2021). For instance, malformations have been observed following exposure to lead (Jin et al., 2019a; He et al., 2020; Ji et al., 2021; Liu et al., 2022), organophosphorus flame-retardant tris (1,3-dichloro-2-propyl) phosphate (Li et al., 2020); triclosan (Wang et al., 2021); propofol (Guo et al., 2015), butylated hydroxyanisole (Baran et al., 2021). Temperature variations can also induce malformations during embryonic development. Incubation of zebrafish embryos at temperatures exceeding 32.5°C from 2.5 until 96 h post-fertilization resulted in various malformations, including head, cardiovascular and tail malformations, spinal deviation, edema and blood accumulations throughout the body (Pype et al., 2015; Cabezas-Sainz et al., 2021).

Analysing neuronal development in zebrafish is greatly facilitated by specific transgenic lines, including tg (elavl3:eGFP), tg (sox10:eGFP), and tg (mbp:mGFP), which aid in identifying alterations in nervous system development (Oh et al., 2022). These alterations may manifest as difference in brain and spinal cord width (Muhsen et al., 2021; Park et al., 2021; Oh et al., 2022), impaired myelination (Guo et al., 2015; Faria et al., 2018; Ji et al., 2021; Park et al., 2021; Wang et al., 2021; Oh et al., 2022), midbrain and hindbrain midline gap defects (Muhsen et al., 2021), pathological changes in various encephalic areas and variation in eye dimensions (Guo et al., 2015; Roy et al., 2016; He et al., 2020; Muhsen et al., 2021; Wang et al., 2021) following exposure to neurotoxic substances. It is worth noting that alteration in apoptotic processes may contribute to some of these malformations.

The impact of the neurotoxic agent can also be evaluated by analysing the expression of genes associated with neuronal development and differentiation (Supplementary Table S2). These genes encompass immature neuronal markers like *nestin* (Chen et al., 2012; Ho et al., 2013; Soares et al., 2017; Qian et al., 2018; Cheng et al., 2020; Xia et al., 2021; Xu et al., 2022a; Xu et al., 2022b; Yu et al., 2022; Xu et al., 2023), as well as mature neuronal markers like growth associated protein 43 (*gap43*) (Chen et al., 2012; Sun et al., 2016; Soares et al., 2017; Faria et al., 2018; Qian et al., 2018; Shi et al., 2018; Jin et al., 2019b; Li et al., 2019; Cheng et al., 2020; Zhu et al., 2020; Chen et al., 2021; Gyimah et al., 2021; Li et al., 2021; Tian et al., 2021; Xu et al., 2021; Xu et al., 2022a; Xu et al., 2022b; Yu et al., 2022; Shi et al., 2023; Xiong et al., 2023; Xu et al., 2023; Zhu et al., 2023), and elav like RNA binding protein 3 (*elavl3*) (Chen et al., 2012; Sun et al., 2016; Qian et al., 2018; Shi et al., 2018; Yang et al., 2019; Zhu et al., 2020; Chen et al., 2021; Gyimah et al., 2021; Wang et al., 2021; Xu et al., 2021; Xu et al., 2022a; Xu et al., 2022b; Fan et al., 2022; Wang et al., 2022; Yu et al., 2022; Xiong et al., 2023; Zhu et al., 2023). Additionally, astrocyte markers such as glial fibrillary acidic protein (*gfap*) (Chen et al., 2012; Sun et al., 2016; Soares et al., 2017; Faria et al., 2018; Li et al., 2018; Qian et al., 2018; Shi et al., 2018; Jin et al., 2019b; Li et al., 2019; Yang et al., 2019; Cheng et al., 2020; Zhu et al., 2020; Bedrossiantz et al., 2021; Chen et al., 2021; Gyimah et al., 2021; Li et al., 2021; Xia et al., 2021; Xu et al., 2022b; Song et al., 2022; Wang et al., 2022; Yu et al., 2022; Zhu et al., 2022; Shi et al., 2023; Xiong et al., 2023), and nerve-related genes such as *mbpa* (Wang et al., 2021; Song et al., 2022), *mef2d* (Wang et al., 2021), *pax6a* (Ho et al., 2013; Li et al., 2019; Wang et al., 2021; Gyimah et al., 2022), *cadm4* (Wang et al., 2021), and markers for secondary motor neurons like *mnx* (Seredick et al., 2012; Muhsen et al., 2021) have been utilised in zebrafish (Supplementary Table S2).

Furthermore, temperature variations exert a neurotoxic effect by modulating the expression of brain protein associated with nervous system development. Proteomic studies conducted on the brain of adult zebrafish have revealed that acute exposition to temperature of 18°C or 34°C leads to changes in the expression of proteins involved in neuronal development (Nonnis et al., 2021), including Map4k4, Ctnnd2, and Gap43 (Nonnis et al., 2021). Furthermore, chronic exposure to 34°C alters the expression of proteins linked to neuronal degeneration and the nervous system (Toni et al., 2019a) such as Pla2g6 (Guo et al., 2018; Jafarzadeh Esfehani et al., 2021), Cacna2d2 (Ivanov et al., 2004; Tedeschi et al., 2016) and Dync1h1 (Kuzma-Kozakiewicz et al., 2013; Mentis et al., 2022).

Among the markers mentioned above, proteomic analysis has shown a reduced expression of Gap43 in both acute treatment at 18°C and 34°C, as well as decrease in Elavl3 expression in 34°C chronic treatment. Conversely, an increase in Gfap and Mbp expression was observed in the chronic treatment at 34°C, while Mbp expression was reduced at 18°C in the chronic treatment. Moreover, the increase of proteins belonging to the Cell adhesion molecules (CADMs) family (Cadm1b, Cadm2a, Cadm3) was observed in acute treatment at 34°C; tubulin beta isoforms were reduced at 34°C in chronic treatment (Tubb5) and increased in acute treatment at 34°C (Tubb1, Tubb2) (Toni et al., 2019a; Nonnis et al., 2021).

## 3 Stress hormones

In some neurotoxicity studies, the whole-body cortisol level is measured to analyse the physiological reaction to stress, which is a valuable parameter for understanding development of any stress due to the toxicity (Biswas et al., 2018) (Figure 2). Cortisol is a primary stress indicator in fishes, which typically increases during exposure to acute or chronic stressors (Ramsay et al., 2006). Cortisol release is regulated by the hypothalamic-pituitary-adrenal axis: corticotropin releasing factor (CRF), secreted by the hypothalamus following perception of a stressor, activates the pituitary to release the adrenocorticotropic hormone which stimulates cortisol release from the adrenal glands (Clark et al., 2011). Elevated cortisol levels have been observed following exposure to various substances, including mercury (II) chloride (HgCl2) (Biswas et al., 2018), acrylamide (Faria et al., 2018), bisphenol (Kim et al., 2022), venlafaxine (Alves et al., 2023) and zinc chloride (ZnCl2) (Sarasamma et al., 2018).

## 4 Proteostasis

Neurotoxic substances can interfere with proteostasis, the complex process vital for maintaining the proteome’s homeostasis (Figure 2). This mechanism encompasses various processes, including chaperone-mediated folding, proteasomal degradation and autophagy, all of which work together to safeguard the integrity and functionality of cells in animals (Motta and Calcagno, 2018). Proteostasis assumes particular significance in long-lived cells such as neurons.

Molecular chaperones play a crucial role in identifying proteins with misfolded conformations that may pose a potential threat to the cell. The primary chaperones belong to the Heat Shock Protein (HSP) family, a group of proteins well known for their roles in protein maturation, refolding and degradation (Miller and Fort, 2018). Some of these chaperones are constitutively expressed, while others are rapidly upregulated in response to stressors like hyperthermia and hypoxia (Miller and Fort, 2018). The main members of this family include Hsp40, Hsp60, Hsp70 and Hsp90 (Fink, 1999). Emerging evidence suggests that these chaperones may influence neurodevelopment by regulating processes such as cell differentiation, neurites outgrowth, cell migration, and angiogenesis (Miller and Fort, 2018). Toxic agents can serve as stressors that modify the expression of HSPs, thereby exposing the cell to potential damage. On one hand, reduced HSP expression can lead to the accumulation of misfolded proteins both inside and outside the cell. On the other hand, increased expression of these chaperones reflects the cell’s attempt to counteract the production of malformed proteins induced by neurotoxic agents. For instance, studies have reported alterations in Hsp expression in teleosts following lead exposure, resulting in reduced Hsp70 and increased Hsp90 expression in zebrafish (Giusi et al., 2008; Li et al., 2019b).

Temperature fluctuations represent a significant stress factor for cells, particularly in heterothermic animals like zebrafish, which lack mechanisms to maintain a constant body temperature. As expected, proteomic analysis of the adult zebrafish brain has shown increased expression of Hsp90 proteins under both low (18°C) and high (34°C) temperature conditions in acute treatments. Additionally, chronic exposure to 18°C led to increased Hsp90 expression. Furthermore, proteins belonging to the Hsp40 and Hsp60 family exhibited increased expression under acute treatment at 34°C (Toni et al., 2019a; Nonnis et al., 2021). Prolonged exposure to both low and high temperatures reduced the expression of proteins involved in protein folding (Toni et al., 2019a).

The ubiquitin-proteasome system (UPS) allows the degradation of specific proteins by the action of the E3 ubiquitin ligase that binds ubiquitin to target proteins, the 26S protein-degrading proteasome, and deubiquitinating enzymes. Proper UPS function is vital for neurons, as its dysfunction can lead to the accumulation of misfolded proteins and the onset of neurodegeneration as observed in Alzheimer’s and Parkinson’s disease (McNaught et al., 2001; Thibaudeau and Smith, 2019). In adult zebrafish, high concentrations of quercetin have been shown to impair proteasome function, resulting in the downregulation of genes associated with the proteasome pathway (Wu et al., 2021). Temperature variation have also been observed to alter the expression of UPS proteins in the zebrafish brain when exposed to both low and high temperatures (Toni et al., 2019a; Nonnis et al., 2021). Specifically, chronic treatment at 18°C and 34°C reduced the expression of UPS proteins such as Psmb7, Psmc1a, and Psmd7 (Toni et al., 2019a).

Autophagy encompasses cellular processes responsible for degrading molecules and organelles within the cell, thereby maintaining a homeostatic balance in protein synthesis, degradation, and organelle turnover (Mizushima and Komatsu, 2011; Liu et al., 2023). Three different types of autophagy have been identified: macro-autophagy, micro-autophagy, and chaperone-mediated autophagy (CMA) (Kaushik and Cuervo, 2012; Martinez-Lopez et al., 2015; Liu et al., 2023). Disruption in autophagic processes can potentially result in neurotoxic effects, either by causing the accumulation and aggregation of misfolded proteins or by degrading proteins and organelles essential for the cell.

Studying the neurotoxic effect on autophagy in zebrafish typically involves analysing the expression of key genes associated with the autophagic process. Notable among these genes are the Autophagy-related (*atg*) genes, such as *atg3*, *atg5* (Li et al., 2018; Li et al., 2020; Li et al., 2021), the Unc-51 like autophagy activating kinase (*ulk*) genes, including *ulk1b* (Li et al., 2021), *ulk2* (Ji et al., 2021). Other critical genes include *becn1* (Li et al., 2018; Li et al., 2020; Li et al., 2021) genes, the *map1lc3a* (Microtubule-associated protein 1A/1B-light chain 3) gene (Liu et al., 2019), and finally the *pink1* (PTEN-induced putative kinase 1) (Li et al., 2020; Li et al., 2021; Yang et al., 2022) and *parkin* (Li et al., 2021; Wang et al., 2021; Yang et al., 2022; Ilie et al., 2022) genes, which are involved in mitophagy (Supplementary Table S2). For example, exposure to silica nanoparticles has been found to increase the expression of *ulk1b*, *beclin 1*, *atg5*, *pink* and *parkin* (Li et al., 2020), while lead exposure resulted in increased *ulk1b* expression and decreased *beclin 1*, *atg5*, and *lc3* expression (Ji et al., 2021).

## 5 Oxidative stress

Metabolism and enzymatic processes generate free radicals both inside and outside the cell. Free radicals are highly reactive molecules characterized by unpaired valence electrons, making them potentially harmful to the cell. They fall into two categories: reactive oxygen species (ROS), including superoxide (O2•−), hydrogen peroxide (H_2_O_2_), hypochlorous acid (HOCl), and reactive nitrogen species (RNS), such as nitric oxide (NO) and peroxynitrite (ONOO-). Mitochondria are the primary source of free radicals within the eukaryotic cell. The transport of electrons in the respiratory chain determines the production and degradation of reactive species and the disruption in this process due to factors like reduced oxygen availability can lead to excessive free radical production. Additionally, inflammatory processes occurring outside cells can generate free radicals through enzymes like myeloperoxidase, xanthine oxidase, and NADPH oxidases. To counteract oxidative stress, aerobic cells employ small molecular weight antioxidants and enzymes, including ascorbate, glutathione, glutathione peroxidase (Gpx), glutathione reductase (Gsr), glutathione-S transferase (Gst), catalase (Cat), superoxide dismutase (Sod), heme oxygenase, peroxidases enzymes, phenolic compounds, and vitamins C and E (Jacob, 1995; Deyashi and Chakraborty, 2016).

Cellular antioxidant mechanisms primarily involve inhibiting free radical production, scavenging free radicals, and enhancing their degradation. Oxidative stress occurs when there is an imbalance between free radical production and elimination. Consequently, increased ROS-producing processes or reduced detoxification mechanisms can contribute to oxidative stress, and this poses a threat to all cells as it can damage vital molecules through lipid peroxidation, protein oxidation, and DNA fragmentation.

The central nervous system is particularly susceptible to oxidative stress due to its high metabolic activity which results in a significant production of ROS, high oxygen consumption, a lack of energy reserves, a high concentration of lipids prone to peroxidation, and elevated levels of iron, all of which act as pro-oxidants (Blomgren and Hagberg, 2006). Scientific evidence suggests a potential link between oxidative stress and the development of neurodegenerative diseases like Alzheimer’s, Parkinson’s, and Huntington’s disease, which are characterized by the formation of neurotoxic protein aggregates in the brain (Alqahtani et al., 2023).

In aquatic organisms, key enzymes for ROS detoxification are Sod, Cat and Gpx (Blomgren and Hagberg, 2006). In zebrafish, the impact of neurotoxic substances on oxidative stress can be studied by analysing the expression and the activity of these enzymes or other markers related to the oxidative stress (Figure 2). For instance, neurotoxicity studies in zebrafish have analysed the gene expression of *sod1* (Powers et al., 2017; Cao et al., 2019; Ding et al., 2023; Zhu et al., 2023), *sod2* (Sarasamma et al., 2018; Cao et al., 2019; Wu et al., 2021; Ji et al., 2021; Patel et al., 2021; Haridevamuthu et al., 2022; Orozco-Hernandez et al., 2022), *cat* (Gu et al., 2021; Ji et al., 2021; Haridevamuthu et al., 2022; Orozco-Hernandez et al., 2022; Kachot and Patel, 2023; Zhu et al., 2023) and *gpx* (*gpx1a* and *gpx4a*) (Ji et al., 2021; Haridevamuthu et al., 2022; Orozco-Hernandez et al., 2022; Ding et al., 2023), *gst* (Haridevamuthu et al., 2022), *gsr* (Haridevamuthu et al., 2022), *gclm* (Wu et al., 2021; Ji et al., 2021; Chian et al., 2022) and *gsto2* (Ji et al., 2021). For example, reduced *sod* expression was observed following treatment with cadmium chloride (Pan et al., 2018), zinc chloride (Sarasamma et al., 2018), mercury chloride (Patel et al., 2021), fipronil (Wu et al., 2021), acrylamide (Haridevamuthu et al., 2022), while lead (Ji et al., 2021) and fluoxetine (Orozco-Hernandez et al., 2022) led to an increase in *sod* expression. Additionally, enzyme activity analysis indicated a decrease in *sod* activity following treatment with photoaged polystyrene (Ding et al., 2023), bisphenol A (Gu et al., 2021), acrylamide (Haridevamuthu et al., 2022), and AlCl3 (Haridevamuthu et al., 2023). Conversely, other neurotoxicity studies reported an increase in *sod* activity following treatment with venlafaxine (Alves et al., 2023), phenytoin (Cardoso-Vera et al., 2022), and lincomycin hydrochloride (Cheng et al., 2020).

Additionally, reduced levels of glutathione (GSH) and alterations in the ratio between glutathione and glutathione disulphide have been analysed as indicators of oxidative stress in zebrafish (Sarasamma et al., 2018; Faria et al., 2019a; Patel et al., 2021; Haridevamuthu et al., 2022) (Supplementary Table S2).

Exposure to thermal fluctuations can trigger oxidative stress in zebrafish by impacting cellular metabolism and influencing the expression of proteins crucial for maintaining redox homeostasis. Adult zebrafish subjected to a temperature drop from 28°C to 12°C experienced a significant elevation in ROS and lipid peroxidation within brain, gill and liver tissues (Wu et al., 2015). This was associated with the activation of an anti-oxidative mechanism, beginning with elevated Cu/Zn-SOD levels, followed by increased *cat* and *gpx* mRNA expressions (Wu et al., 2015). Moreover, Tseng and collaborators demonstrated an increase in cellular protein carbonyl concentration in zebrafish brain after cold exposure from 28°C to 18°C (Tseng et al., 2011). This exposure also led to increased Sod activity and gene expression of *cat*, uncoupling proteins (*zucp 2–5*) and homologous of peroxisome proliferator-activated receptor (Tseng et al., 2011). In the brain of adult zebrafish subjected to 21 days of exposure to 34°C, a reduction in the expression of proteins associated with cellular redox homeostasis, oxidoreductase activity, and mitochondrial respiratory chain complex 1, including Prdx2, Glrx, Cat, Gpx1a was observed (Toni et al., 2019a). Furthermore, acute treatment led to a decrease in Gst expression. Chronic exposure at both 18°C and 34°C resulted in the reduced expression of Gpx and Gsto2, while Cat expression decreased specifically at 34°C (Toni et al., 2019a; Nonnis et al., 2021).

## 6 Mitochondria

Nerve cells exhibit a robust cellular metabolism and heavily rely on the adenosine triphosphate (ATP) generated within their mitochondria to uphold resting membrane potentials and facilitate the firing of action potentials (Howarth et al., 2012). Consequently, disturbances in mitochondrial functions are closely linked to neuron dysfunction, neuronal death and various neurological disorders.

Due to this vulnerability, nerve cells are particularly sensitive to neurotoxic substances that interfere with mitochondria activity, particularly within the respiratory chain. Mitochondria, once considered isolated and static organelles, are now understood as dynamic and interactive networks. Their quantity and size can change through fusion, fission and mitophagy processes, driven by energy demands or cell damage (Scott and Youle, 2010). Effective energy production depends not only on mitochondrial function but also on their cellular placement. Numerous mitochondria are present in the axon terminal and synaptic button to cope with the high energy requirements. Furthermore, mitochondria play a pivotal role in regulating intracellular calcium concentration, especially crucial at presynaptic terminals for the regulation of neurotransmission (Billups and Forsythe, 2002; Kwon et al., 2016; Marland et al., 2016; de Juan-Sanz et al., 2017; Vaccaro et al., 2017). The proper distribution of mitochondria rely on axonal transport which allows their movement from the cell body to the axon terminal (Seager et al., 2020). Consequently, the neurotoxic agent has the potential to affect mitochondria by altering their morphology, localization and molecular processes (Figure 2).

Electron microscopy has revealed morphological alterations in mitochondria in neurodegenerative pathologies such as amyotrophic lateral sclerosis (Atsumi, 1981; Sasaki and Iwata, 2007). Studies conducted in teleost models exposed to neurotoxic substances have shown observable alterations, including lesions, vacuolization, damage to the outer membrane, loss of cristae, and ultimately swollen and deformed morphology (Sheng et al., 2016; Ren et al., 2018; Li et al., 2019a).

Deficiencies in the respiratory chain result in reduced ATP production, oxidative stress generation, and are linked to neurodegenerative diseases such as Alzheimer’s disease, Parkinson’s disease, Huntington’s disease, amyotrophic lateral sclerosis (Golpich et al., 2017), spinocerebellar ataxia (Lax et al., 2012), and other peripheral neuropathies (Rizzo et al., 2016). The neurotoxic impact on mitochondria can also be assessed by analysing the expression of genes encoding mitochondrial complexes, their activity (Li et al., 2019), mitochondrial membrane potential and ATP content (He et al., 2020). Reduction of respiratory complex activity was observed following treatment with pyraclostrobin (Li et al., 2019) and propofol (He et al., 2020).

Temperature variations significantly affect brain mitochondria in adult zebrafish. Proteomic analyses conducted on adult zebrafish’s brain have demonstrated alteration in the expression of several proteins involved in the electron transport chain and oxidative phosphorylation under both low and high temperatures (Toni et al., 2019a; Nonnis et al., 2021). Furthermore, the reduction of proteins related to mitochondrial transport was observed at 34°C, suggesting impaired mitochondrial positioning in synaptic terminals (Li et al., 2004), where they play pivotal roles in regulating neurotransmission (Billups and Forsythe, 2002; Medler and Gleason, 2002; David and Barrett, 2003; Talbot et al., 2003) and synaptic plasticity (Levy et al., 2003; Kang et al., 2008; Toni et al., 2019a). Both acute and chronic exposure to low temperatures resulted in reduced expression of brain proteins involved in oxidative phosphorylation. Furthermore, chronic treatment at 18°C led to decreased expression of ATP synthesis coupled proton transport. Exposure to high temperatures also impacted mitochondria, resulting in a reduced expression of proteins associated with oxidative phosphorylation and mitochondrial respiratory chain complex I (Toni et al., 2019a).

## 7 Morphology and characteristics of neurons

In zebrafish, the assessment of neurotoxic effects involves the analysis of neuronal morphology and characteristics within various cell populations. For instance, motoneurons identifiable by green fluorescent protein (GFP) expression and primary sensory neurons like Rohon-Beard (RB) neurons can serve as valuable indicators (Cuevas et al., 2013).

Neurotoxicity studies have unveiled ultrastructural changes in perikaryon encompassing alteration in intercellular junctions, cell membrane blebbing, cytoplasmic edema, mitochondrial alteration, expansion of the endoplasmic reticulum, nuclear deformation, widening of the nuclear membrane, and modifications in the Nissl bodies (Figure 2). Aberrant expression of vesicular markers, such as *lamp1l* (lysosomal-associated membrane glycoprotein 1-like) and *sara2* (SAR1a gene homologue) has been observed (Ho et al., 2013).

Following neurotoxic treatments, analysis of axons has unveiled severe phenotypes characterized by vacuolar degeneration of axonal ending, truncation at the horizontal myoseptum, innervation of neighbouring myotomes, missing axons, ectopic branches, and dissolution of the myelin sheath (Muhsen et al., 2021).

Gene expression analysis related to axons has demonstrated alteration in the expression of regeneration-associated genes such as growth-associated protein Gap-43 (*gap43*) (Chen et al., 2012; Sun et al., 2016; Soares et al., 2017; Faria et al., 2018; Qian et al., 2018; Shi et al., 2018; Jin et al., 2019b; Li et al., 2019; Cheng et al., 2020; Zhu et al., 2020; Chen et al., 2021; Gyimah et al., 2021; Li et al., 2021; Tian et al., 2021; Xu et al., 2021; Xu et al., 2022a; Xu et al., 2022b; Chang et al., 2022; Yu et al., 2022; Shi et al., 2023; Xiong et al., 2023; Xu et al., 2023; Zhu et al., 2023) and *α1 tubulin* (Sun et al., 2016; Li et al., 2018; Shi et al., 2018; Yang et al., 2019; Fu et al., 2020; Zhu et al., 2020; Chen et al., 2021; Gyimah et al., 2021; Xu et al., 2021; Xu et al., 2022a; Xu et al., 2022b; Heredia-García et al., 2023; Shi et al., 2023; Xu et al., 2023). Additionally, nerve related genes like *pax6a* (Ho et al., 2013; Li et al., 2019; Wang et al., 2021; Gyimah et al., 2022), *elavl3* (Chen et al., 2012; Sun et al., 2016; Qian et al., 2018; Shi et al., 2018; Yang et al., 2019; Zhu et al., 2020; Chen et al., 2021; Gyimah et al., 2021; Wang et al., 2021; Xu et al., 2021; Xu et al., 2022a; Xu et al., 2022b; Fan et al., 2022; Wang et al., 2022; Yu et al., 2022; Xiong et al., 2023; Zhu et al., 2023) and *ncam1a*, along with axon formation gene like *mbpa* (Song et al., 2022) have also shown altered expression following neurotoxic treatments (Supplementary Table S2).

Brain proteomic analysis suggests that thermal variations can also impact neurons, axons and dendrites. Proteomic analyses have unveiled altered expression of protein networks associated with dendritic and neuritic growth and branching at both 18°C and 34°C under acute and chronic conditions (Toni et al., 2019a; Nonnis et al., 2021). Notable proteins affected include Srcin1 (Calipari et al., 2019), Sipa1l1 (Pak et al., 2001; Dolnik et al., 2016), and Ryr2 (Bertan et al., 2020).

At the synaptic level, neurotoxicity can disrupt the expression of synaptic vesicle cycling genes such as synapsin 2a (*syn2a*) (Chen et al., 2012; Sun et al., 2016; He et al., 2017; Faria et al., 2018; Li et al., 2018; Qian et al., 2018; Shi et al., 2018; Jin et al., 2019b; Li et al., 2019; Zhu et al., 2020; Gu et al., 2021; Tian et al., 2021; Xu et al., 2022a; Xu et al., 2022b; Gyimah et al., 2022; Haridevamuthu et al., 2022; Wang et al., 2022; Yu et al., 2022; Shi et al., 2023; Xiong et al., 2023), syntaxin binding protein 1b (*stxbp1b*), synaptotagmin 1a (*syt1a*) (Faria et al., 2018; Haridevamuthu et al., 2022), and *nsfa* (N-ethylmaleimide-sensitive factor a) (Faria et al., 2018) (Supplementary Table S2). For instance, reduced *syn2a* expression has been observed following treatment with olaquindox (Guo et al., 2021), methamidophos (He et al., 2017), lead (Li et al., 2019), and TDCPP (Wang et al., 2015).

Thermal alterations have also been shown to affect the expression of proteins associated with synaptic vesicles and their transport. Zebrafish brains exhibited reduced expression of synapse-associated proteins following acute and chronic thermal treatments at both 18°C and 34°C (Nonnis et al., 2021), influencing synaptic-related proteins as well (Toni et al., 2019a; Nonnis et al., 2021).

## 8 Neurotransmission

Zebrafish serves as an excellent model for studying the neurotransmitter system due to the overall organisation of its nervous system similar to humans and the presence of comparable neurotransmitter systems, including glutamatergic, cholinergic, serotonergic, dopaminergic, adrenergic, GABAergic, and histaminergic systems (Babin et al., 2014; Horzmann and Freeman, 2016; Prats et al., 2017; Faria et al., 2018). For instance, exposure of zebrafish to acrylamide for 3 days resulted in altered expression of acetylcholine (Ach), serotonin, dopamine, norephinephrine and aspartate (Faria et al., 2018). The neurotoxic action appears complex since acrylamide determines different effects on larvae and adults (Prats et al., 2017; Faria et al., 2018).

Neurotoxic substances can disrupt neurotransmission, and their effects can be studied by examining neurotransmitter levels in the nervous system, the gene and protein expression of neurotransmitter receptors, as well as the expression and activity of enzymes involved in neurotransmitter synthesis or degradation, along with other critical proteins involved in neurotransmission (Figure 2).

Glutamate, the principal excitatory neurotransmitter in the nervous system, may play a role in neurotoxic events through various mechanisms. Excessive activation of glutamate receptors can lead to neuronal death, a phenomenon known as “excitotoxicity” (Zhou and Danbolt, 2014). Glutamate can also induce calcium homeostasis disruption and mitochondrial dysfunction in neurons (Khodorov, 2004), along with oxidative stress and apoptosis (Parfenova et al., 2006). Studies in zebrafish underscore the significance of glutamate signalling in the myelination process (Turan et al., 2021). Toxic substances, such as ethanol, can alter glutamate uptake, resulting in astrogliosis and neuroinflammation in the adult zebrafish brain (Vizuete et al., 2022). Exposure to TBBPA-DHEE (Feng et al., 2023) and acrylamide (Faria et al., 2018) has been shown to reduce glutamate content in the zebrafish brain. Evidence suggests that alterations in glutamate levels in zebrafish can be associated with behavioural impairments (Da Silva Lemos et al., 2022).

Concerning the cholinergic system, the neurotransmitter acetylcholine (Ach), synthesized by the enzyme choline acetyl transferase (ChAT), is released into the synaptic cleft and rapidly degraded by acetyl choline esterase (AchE) and butyrylcholinesterase into acetic acid and water (Dal Forno et al., 2013). Dysregulation of Ach, ChAT, and AchE has been widely utilised as indicators of neurotoxicity in zebrafish (Agostini et al., 2018). Neurotoxicity studies have revealed alterations in Ach levels. For example, exposure to acrylamide reduced Ach levels in the adult brain but increased levels in the whole-larvae (Faria et al., 2018). Increases in Ach levels were also observed in zebrafish larvae following exposure to hexabromobenzene and pentabromobenzene (Chen et al., 2021). The interference with AchE is a significant pathway through which chemical substances induce neurotoxicity (Reddy et al., 2007; Jafarzadeh et al., 2022). For instance, exposure to fluoxetine or a combination of microcystin-LR and nitrite significantly decreased AchE expression and activity in zebrafish brain (Yang et al., 2022; Orozco-Hernandez et al., 2022). Additionally, exposure to waterborne microplastics led to increased AchE and ChAT activity in the zebrafish brain (Yu et al., 2022).

The neurotoxic effects on the GABAergic system are typically investigated in zebrafish by analysing the expression of genes associated with the GABAergic pathway, such as GABA synthesizers [*gad1b* (Cheng et al., 2020; Shi et al., 2022; Yu et al., 2022; Ding et al., 2023; Xiang et al., 2023) and *gad2* (Ding et al., 2023)], receptors (*gabra1* (Shi et al., 2022; Liu et al., 2023; Ding et al., 2023), *gabra6a* (Atzei et al., 2021), *gabra6b* (Liu et al., 2023), *gabrb3* (Luo et al., 2023), *gabrg2* (Ding et al., 2023; Xiang et al., 2023)), and transporters (*gat1* (Cheng et al., 2020; Ding et al., 2023; Xiang et al., 2023), *gat3*, *vgat*). For example, exposure to lead has been shown to increase *gad1b* and *gad2* expression and modulate the expression of *gabra1*, *gabbr1a*, *gat1*, *gat3*, *vgat* expression (Wirbisky et al., 2014) (Supplementary Table S2). Additionally, the levels of GABA can be analysed (Tian et al., 2021).

In teleost fish, including zebrafish, the dopaminergic system is present, although there are differences compared to mammals. Dopaminergic neurons are not located in the midbrain, but are found in the paraventricular organs, the periventricular nucleus of the posterior tuberculum, and the posterior tuberal nucleus (Rink and Wullimann, 2001; 2004). Studies suggest that the periventricular nucleus of the posterior tuberculum in fish is equivalent to the A9 and A10 neurons of mammals (Yamamoto and Vernier, 2011). The impact on the dopaminergic system can be investigated by analysing the morphology of dopaminergic neurons (Cui et al., 2013; Yao et al., 2017; Jin et al., 2019a; Wang et al., 2019), aided by the availability of zebrafish transgenic lines with GFP-labelled dopaminergic neurons. Analysis of dopamine levels (Wang et al., 2015; Sheng et al., 2016; Soares et al., 2017; Wang et al., 2019; Han et al., 2021; Yang et al., 2022) and its metabolite, hydroxy-phenyl acetic acid, along with the expression of dopamine transporter (*dat*) genes (Ji et al., 2021; Gyimah et al., 2022), dopamine receptors (*drd1b* (Li et al., 2019; Tran et al., 2021; Hong et al., 2022; Liu et al., 2023), *drd2a* (Cao et al., 2019; Gyimah et al., 2022; Shi et al., 2022; Liu et al., 2023; Ding et al., 2023; Mahapatra et al., 2023; Torres-Ruiz et al., 2023; Xiang et al., 2023), *drd2b* [Shi et al., 2022; Liu et al., 2023; Torres-Ruiz et al., 2023)] and developmental genes correlated to dopamine such as *manf* (Li et al., 2018; Qian et al., 2018; Li et al., 2020; Zhu et al., 2020; Tian et al., 2021; Hong et al., 2022; Heredia-García et al., 2023; Shi et al., 2023), *otpa*, *otpb* (Wang et al., 2019), *wnt1* (Ho et al., 2013), *wnt5b* (Ho et al., 2013), *wnt10b* (Xu et al., 2022a), *wnt4a* (Xu et al., 2022a), *wnt3a*, *wnt5a* (Wang et al., 2019) can be analysed (Supplementary Table S2). Treatment with lead in zebrafish larvae significantly reduced the number of dopaminergic neurons in the midbrain (Jin et al., 2019b). Furthermore, exposure to substances such as graphene oxide or to microcystin-LR and nitrite reduced *dat* gene expression and dopamine levels (Soares et al., 2017; Yang et al., 2022).

Proteomic analyses of the adult zebrafish brain suggest that alteration in temperature may affect neurotransmission. Both acute or chronic exposure to low temperatures have been associated with reduced expression of opioid-associated proteins, adrenergic, metabotropic-glutamate and 5-HT signalling (Toni et al., 2019a). Additionally, chronic exposure to both 18°C and 34°C has been found to reduce EGF/FGF signalling (Toni et al., 2019a), while the expression of AchE increases in response to acute treatment at 18°C and at 34°C (Toni et al., 2019a).

Neurotoxicity studies in zebrafish indicate that exposure to various substances, including drugs, nanoparticles, and environmental contaminants, can disrupt neurotransmitter systems, potentially leading to neurobehavioural alterations.

## 9 Behaviour

Behavioural change can serve as indicator of alterations in the functioning of the nervous system, making behavioural tests valuable tools in neurotoxicity studies. Teleost fish behaviour can be studied both in their natural environment and in laboratory settings (Malavasi et al., 2013; Manciocco et al., 2015; Butler et al., 2022). Zebrafish has gained recognition as an excellent model in neuroscience, with several behavioural tests applicable to both larval and adult stages.

Behavioural tests involve the analysis of fish positioning, preference for specific areas in the tank or apparatus, and locomotor parameters such as distance travelled, speed, meandering and freezing, often using specialised software programs (Figure 2). Commonly analysed parameters in larval behavioural tests include total time of motion, distance travelled, speed, acceleration, freezing in light or dark conditions. Many neurotoxic substances, including acrylamide (Park et al., 2021), 6-hydroxydopamine (Zhang et al., 2017), 3,4-methylenedioxyamphetamine (Tombari et al., 2023), fenpropathrin (Yu et al., 2022) have been shown to reduce larval motor activity.

**FIGURE 3:**
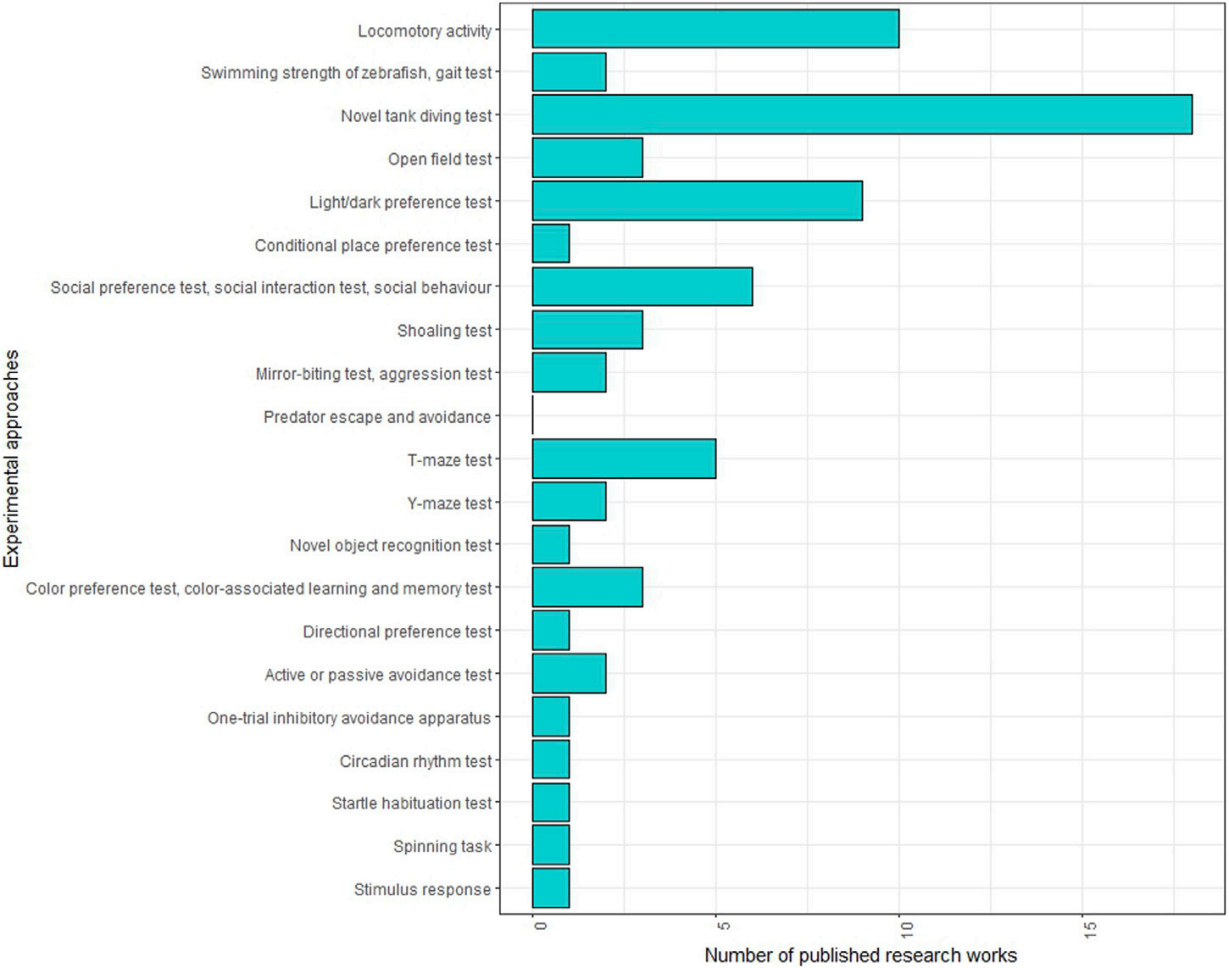
Frequencies of behavioural tests conducted on adult zebrafish in neurotoxicity studies in the literature.

Several behavioural tests are employed in adult zebrafish studies (Figure 3; Supplementary Table S3). One of the most commonly used tests in neurotoxicity investigations is the novel tank test (NTT), which assesses fish behaviour in a new environment. This test can determine whether the fish exhibits anxiety-related behaviour, characterised by increased freezing events and time spent at the tank’s bottom, or boldness behaviour, indicated by more time spent and greater distance covered in the upper tank area. The specific behaviour observed may depend on the neurotoxic agent used. For instance, a reduction in time spent in the upper area of the tank was observed following the administration of metformin (Elizalde-Velazquez et al., 2022a), methamphetamine (Bedrossiantz et al., 2021), acrylamide (Faria et al., 2018; Faria et al., 2019b) and silica nanoparticles (Li et al., 2020), while an increase in upper area exploration was observed with phenytoin (Cardoso-Vera et al., 2022) and guanylurea (Elizalde-Velazquez et al., 2022b). Interestingly, thermal treatments also influenced zebrafish behaviour in NTT, showing opposite effects at low and high temperatures. Time spent in the surface area decreased in response to acute exposure at 18°C but increased with chronic treatment at 34°C.

The dark and light preference test is another widely used test that measures scototaxia. Adult zebrafish naturally tend to prefer dimly lit and sheltered areas, and neurotoxic actions can lead to either a reduction or an increase in the preference for the dark area. For example, methamphetamine (Bedrossiantz et al., 2021) and bisphenol A increased the time spent in the light area (Thayumanavan et al., 2022), while silica nanoparticles reduced it (Li et al., 2020). Interestingly, low and high temperatures had opposing effects on scototaxia: acute treatment at 18°C increased preference for the dark area, while chronic treatment at 34°C decreased it.

The social preference test capitalized on the natural inclination of zebrafish to engage in group interactions (Gerlai et al., 2000; Saverino and Gerlai, 2008) and assesses their preference for conspecifics. Neurotoxic effects can manifest as alterations in the subject’s interest in conspecifics, as evidenced by exposure to substances like methamphetamine (Bedrossiantz et al., 2021), organophosphorus flame-retardant tris (1,3-dichloro-2 -propyl) phosphate (Li et al., 2020), venlafaxine (Alves et al., 2023), which reduced the time spent in the social area. Similar behavioural changes were observed in zebrafish exposed to 34°C for 21 days, resulting in reduced time spent and distance covered in the social area.

The mirror biting test measures aggressive behaviour and can be employed in conjunction with other tests to evaluate the neurotoxic effects of chemicals. Changes in nervous system function can either increase or decrease aggressiveness, as aggression is a complex behaviour influenced by external stimuli, primarily visual cues, their interpretation and subsequent motor responses. For example, the administration of paraquat to adult zebrafish increased aggression, measured as a higher number of aggressive episodes (Müller et al., 2018), while treatment with zinc chloride reduced aggression, indicated by reduced mirror biting time (Sarasamma et al., 2018). Notably, variations in temperature can also affect fish aggressivity, as chronic exposure to 34°C reduced the number of mirror bites.

T-maze and Y-maze tests are utilised to evaluate complex cognitive processes like spatial memory and learning. They involve consecutive trials to assess how the environmental exploration is influenced by past experience, providing insights into learning and memory capability. These tests have proven effective in neurotoxicity studies. For example, T-maze test revealed reduced spatial working memory performance in adult zebrafish exposed to lead (Li et al., 2019), silica nanoparticles (Li et al., 2020), benzophenone 1 (Song et al., 2022) and neurotoxin β-N-methylamino-L-alanine (Wang et al., 2020). Similarly, TiO2 nanoparticles treatment reduced spatial exploration, recognition, and interest in a new environment in the Y-maze test (Sheng et al., 2016). Furthermore, both acute and chronic treatment at 18°C and 34°C impaired new environment exploration and spatial memory in adult zebrafish subjected to the Y-maze test (Toni et al., 2019a; Nonnis et al., 2021).

## 10 BDNF involvement in chemical- and temperature-induced neurotoxicity

BDNF is a well-known neurotrophic factor that exerts its effect by binding to Tropomyosin receptor kinase B (TrkB) and p75NTR receptors, and accumulating experimental evidence suggests its involvement in neurotoxicity. BDNF is highly conserved throughout vertebrate evolution, with zebrafish and human BDNF sharing a 91% sequence identity (Lucini et al., 2018). Additionally, akin to mammals, BDNF is widely expressed within the zebrafish brain (Hashimoto and Heinrich, 1997; De Felice et al., 2014; Nittoli et al., 2018). The available evidence strongly indicates that BDNF functions are conserved between zebrafish and mammals (Cacialli et al., 2016), underscoring zebrafish’s suitability as model for studying BDNF. While most information regarding BDNF comes from mammalian studies, the last 15 years have witnessed a growing number of BDNF investigations conducted in zebrafish (Lucini et al., 2018; Aragona et al., 2022), leading to the creation of mutant BDNF^−/−^zebrafish (D'Agostino et al., 2022; Maffioli et al., 2022).

Notably, BDNF may be play a pivotal role in many of the neurotoxicity-related aspects discussed earlier. Concerning neuronal development, BDNF is essential for neurogenesis, neuronal differentiation, survival, growth and plasticity (Lindholm et al., 1996; Zigova et al., 1998; Pencea et al., 2001; Lee et al., 2002; Chan et al., 2008; Bawari et al., 2019). Emerging evidence suggests that BDNF regulates autophagy in neurons and axons (Chen et al., 2013; Kononenko et al., 2017; Nikoletopoulou et al., 2017; Sidibe et al., 2022). Furthermore, studies on BDNF-deficient mice (Hacioglu et al., 2016) and schizophrenia patients (Zhang et al., 2015; Wei et al., 2020) suggest a direct interplay between BDNF levels and oxidative stress in the brain. BDNF may also influence neuron morphology as it can act as both autocrine and paracrine signal, triggering neurite differentiation into axons (Shelly et al., 2007; Cheng et al., 2011) and increasing dendritic spine density (Alonso et al., 2004; Von Bohlen Und Halbach and Von Bohlen Und Halbach, 2018). BDNF is also implicated in neurotransmission by promoting synaptic plasticity (Bawari et al., 2019) and influencing neurotransmitter release (Sala et al., 1998; Tyler and Pozzo-Miller, 2001). BDNF’s involvement in axonal transport, the cellular process crucial for delivering essential cargoes within neurons, is noteworthy. Malfunctions in axonal transport can disrupt neuronal cell biology and lead to neurodegenerative pathologies (Tosolini et al., 2022). Experimental evidence showed that BDNF play a role in promoting anterograde transport of TrkB (Cheng et al., 2011) and in regulating mitochondrial distribution (Yoshii and Constantine-Paton, 2010; Su et al., 2014). BDNF’s influence on mitochondria extends further as it enhances mitochondrial respiratory coupling at complex I (Markham et al., 2004) and augments mitochondrial energy production (Burkhalter et al., 2003). Consistently, increased BDNF expression was detected in response to environmental factors demanding higher cellular energy, such as cognitive stimulation and exercise (Mattson et al., 2004; Cheng et al., 2010).

Given the crucial functions regulated by BDNF in the nervous system, it comes as no surprise that altered BDNF expression can impact animal behaviour. In mice and rat, disrupted BDNF expression has been linked to locomotor activity, eating behaviour (Kernie et al., 2000), and social behaviour (Berry et al., 2015). Furthermore, correlations between BDNF levels and anxiety or depression have been observed (Martinowich et al., 2007; Zhang et al., 2020), with increasing evidence suggesting an association between low BDNF expression and suicidal behaviour (Dwivedi, 2009).

Accumulating evidence suggest that BDNF may play a crucial role in neurotoxic events, as exemplified by neurotoxic effects of methamphetamine (Jayanthi et al., 2021), which reduces BDNF levels. Such reduction in BDNF levels has been associated with neurodegenerative events in the rat hippocampus (Wang et al., 2018; Malczynska et al., 2019; Shahidi et al., 2019; Salehzadeh et al., 2020).

Given these findings, heterozygous (BDNF^+/−^, HT) and knock-out (BDNF^−/−^, KO) animal models represent valuable tools for studying neurotoxicity. KO mice exhibit early postnatal lethality (Ernfors et al., 1994). More recently, HT and KO zebrafish models have been developed, offering a promising teleost-based approach to investigate the neurotoxic effects associated with this neurotrophin. Interestingly, KO adult zebrafish are viable and capable of reproduction, providing a unique opportunity to explore BDNF’s role across various developmental stages.

The transcriptome analysis of KO larvae have unveiled altered gene expression patterns related to nervous system development, cell morphogenesis, axon and dendrite development, neurotransmitter transport and secretion, as well as synaptic organisation (D'Agostino et al., 2022). Further examination of the cerebral proteome in adult KO zebrafish has confirmed significant alteration in protein expression profiles attributable to neurotoxicity (Maffioli et al., 2022). These results point to reduced expression of protein networks associated with neuronal development and morphology. Additionally, the diminished expression of proteins involved in the ubiquitin proteasome pathway suggests compromised proteostasis capacity (Maffioli et al., 2022).

Proteomic analysis has also revealed that the absence of BDNF strongly impacts neurotransmission, leading to reduced expression of proteins within signalling pathways for several neurotransmitter systems, including histaminergic, GABAergic, dopaminergic, adrenergic, serotonergic, cholinergic, glutamatergic pathways. Furthermore, a decrease in FGF and EGF signalling pathways has been observed (Maffioli et al., 2022). Notable among the downregulated proteins in KO zebrafish are *abat* (4-Aminobutyrate Aminotransferase), *c3a*.*1* (Complement C3a Receptor 1), *c3a*.*2* (complement C3a, tandem duplicate 2), *camk2a* calcium/calmodulin-dependent protein kinase II alpha, *glsa* (Glutaminase), *gpx1a* (Glutathione Peroxidase 1), *mao* (Monoamine Oxidase), *nsfa* (N-ethylmaleimide-sensitive factor a), *sod2* (Superoxide Dismutase 2), *syt1a* (Synapto-tagmin 1), corresponding to genes listed in Supplementary Table S2.

Moreover, BDNF deficiency-induced neurotoxic effects extend to zebrafish behaviour in both larvae (D'Agostino et al., 2022; Lucon-Xiccato et al., 2023) and adults (Maffioli et al., 2022). KO larvae and adults display higher levels of locomotor activity and reduced scototaxia, indicating reduced anxiety levels. Additionally, KO zebrafish exhibit greater exploration of the top area of the tank during the novel tank test, underscoring their increased boldness. Finally, KO zebrafish show reduced cognitive abilities as detected by the Y-maze test. The role of BDNF in temperature-induced neurotoxicity in zebrafish is also noteworthy, as exposure to 34°C results in reduced BDNF gene expression and altered neurotoxicity-related protein expression in HT and KO zebrafish (Maffioli et al., 2022).

## 11 Combined effect of temperature and toxic substances

Many aquatic organisms are heterothermic and they often encounter multiple environmental stressors simultaneously in their natural habitats. These stressors can interact to produce adverse effects on these organisms. Standard ecotoxicity tests are typically conducted under optimal conditions, including the species’ specific standard temperature, and they do not provide insights into how temperature interacts with other toxic agents.

Temperature is a fundamental abiotic parameter significantly influencing animal life. As such, numerous studies have explored the combined effects of temperature changes and exposure to various toxic substances, including metals, pesticides, and natural toxins in invertebrates and fish (Cairns et al., 1975; Heugens et al., 2001). These studies have shown that chemical toxicity tends to increase with rising temperatures, often coinciding with elevated metal accumulation (Heugens et al., 2001).

However, there has been a limited number of studies specifically focused on zebrafish, with a primary focus on the effects of metals such as zinc, copper, and cadmium, and less emphasis on neurotoxic effects. Research involving zebrafish has indicated that exposure to cadmium in conjunction with temperature stress can result in a synergistic toxic effect. For instance, severe consequences for developing embryos exposed simultaneously to cadmium ions and cold stress (21°C) were observed, including increased mortality, a substantial reduction in average heart rate, and decreased embryo hatchability (Hallare et al., 2005). Similarly, Guo and collaborators found that combined exposure to elevated temperature and cadmium led to increased lipid peroxidation, ultrastructural damage in the liver, and elevated mortality (Guo et al., 2018). Vergauwen and collaborators further emphasized that the interaction between temperature and cadmium toxicity became increasingly pronounced as temperature increased (Vergauwen et al., 2013). Furthermore, studies have uncovered significant interactions between copper and increasing temperature, resulting in higher embryo mortality (Dorts et al., 2016). Tests focusing on the early development of zebrafish embryos and larvae have also demonstrated a synergistic effect of elevated temperature and nickel exposure (Scheil and Kohler, 2009). In summary, a growing body of research highlights the critical importance of considering temperature as a key factor when evaluating the toxicity of various agents in aquatic organisms, particularly zebrafish.

## 12 Conclusion

Zebrafish serves as an excellent model for investigating chemical- and temperature- induced neurotoxicity due to its amenability for rapid analyses spanning from molecular to behavioural assessment across all developmental stages. The study of neurotoxicity is inherently intricate, stemming from a myriad of interconnected cellular events and mechanisms that can mutually exacerbate one another, ultimately setting off a cascade of damage within the nervous system. The findings presented in this work underscore the pivotal role of mitochondria in neurotoxic events (Figure 2).

As reported above, mitochondria serve as the primary source of ATP production, which is critical for energy-demanding cells such as neurons. Consequently, mitochondrial dysfunction can have a cascading effect on various other cellular processes that are integral to neurotoxicity. Mitochondria contribute significantly to the production of ROS during the electron transport chain, where a fraction of electrons can prematurely escape and interact with molecular oxygen and other molecules, giving rise to ROS, such as the superoxide anion and hydrogen peroxide. These ROS can, in turn, inflict damage on mitochondria and other cellular components (Kowalczyk et al., 2021). Moreover, the limited availability of ATP for enzymes such as glutathione reductase limits the antioxidant response (Figure 2A).

Mitochondrial dysregulation affects cytosolic proteostasis by regulating the synthesis, stability, and degradation of proteins (D'Amico et al., 2017). Insufficient energy availability hampers proper proteostasis, as both the UPS and the autophagic process depend on ATP for effective functioning. Proteostasis mechanisms, through mitophagy, on the other hand, can regulate the number of mitochondria present in the cell (Ding and Yin, 2012) (Figure 2B).

Furthermore, an intricate interplay exists between oxidative stress and proteostasis, as one of the early responses to excessive ROS is the induction of mitophagy, which can reduce oxidative damage and ROS production (Shefa et al., 2019). Multiple lines of evidence have shown that ROS interacts with both ubiquitin-dependent and receptor-dependent mitophagy pathways (De Gaetano et al., 2021; Puente-Bedia et al., 2022) (Figure 2C).

Mitochondria also play a crucial role in embryonic development by undergoing structural and positional changes that enable them to provide the necessary energy for the embryo (Harvey, 2019; May-Panloup et al., 2021) (Figure 2D) and influence the morphology of neurons (Figures 2E, F). In fact, reduced energy availability impairs the functionality of the cytoskeleton and motor proteins, affecting the shape, organelle positioning, and maintenance of dendrites, axons, and synapses. Impaired synaptic vesicle transport and exocytosis processes can further disrupt neurotransmitter release (Guo et al., 2017; Rangaraju et al., 2019) (Figure 2G). Moreover, the development can also be affected by autophagy (Aburto et al., 2012) (Figure 2H) and oxidative stress (Guerriero and D'Errico, 2022) (Figure 2I).

Altered neuronal and synaptic morphology can influence neurotransmission (Udvary et al., 2022) and neurotransmission, in turn, can affect axon morphology (Rudiger and Bolz, 2008) (Figure 2J).

Neurotransmission can also influence oxidative stress; for example, excessive glutamate release and subsequent glutamatergic neuronal stimulation increase the production of ROS, which, in turn, induces oxidative stress, excitotoxicity, and neuronal damage (Savolainen et al., 1998). Moreover, oxidative stress can affect neurotransmission (Dalvi-Garcia et al., 2021) (Figure 2K).

Cortisol plays a pivotal role in the response to stressful conditions, also influencing the functioning of mitochondria (Roosevelt et al., 1973; Picard and McEwen, 2018) (Figure 2L) and oxidative stress (Nakano et al., 2014; Espinoza et al., 2017; Mukai et al., 2022) (Figure 2M).

The effects of neurotoxic agents can, therefore, be complex and may result in behavioural alterations through the reciprocal influence of mitochondria (Picard and McEwen, 2018; Zhang et al., 2020) (Figure 2N), cortisol (Overli et al., 2002; Hill et al., 2008; Bagner et al., 2010; Sloman, 2010; Caplin et al., 2021) (Figure 2O), and above all neurotransmission (Meeusen and De Meirleir, 1995; Scerbina et al., 2012; Maximino et al., 2013; Wang et al., 2019) (Figure 2P).

In conclusion, the exploration of neurotoxicity is an intricate endeavour, marked by the multifaceted nature of the processes involved and their intricate interconnections. To probe this intricate domain effectively, it is imperative to employ robust study models, and zebrafish emerge as a promising candidate for this purpose. While a plethora of markers and experimental approaches exist for neurotoxicity investigations, the establishment of standardized analysis protocols is paramount. These protocols will enable researchers to attain lucid, comprehensive, and readily comparable results across various studies. It is crucial to acknowledge that neurotoxic effects can be provoked not only by chemical substances but also by fluctuations in environmental temperature. The neurotoxic consequences stemming from temperature variations have the potential to augment the impact of dissolved chemical agents, even when present in low concentrations. Given the continuous influx of both natural and anthropogenic pollutants into aquatic ecosystems, coupled with the amplified thermal variations attributed to global warming, fish populations are exposed to heightened risks of neurotoxicity. Future investigations should therefore focus on delving deeper into the neurotoxic effects stemming from temperature variations and elucidating the synergistic effects that result from the interplay between thermal fluctuations and chemical neurotoxic agents. These studies will undoubtedly contribute valuable insights to our understanding of neurotoxicity and its implications for aquatic ecosystems.

## Supplementary material

SUPPLEMENTARY TABLE S1 Experimental approaches adopted in the main works analysing the neurotoxic effect of specific treatments in zebrafish. The search was conducted in the Pubmed database (https://pubmed.ncbi.nlm.nih.gov/) on 06/19/2023 by entering the following criteria [Danio rerio (Title) and neurotox* (Title) and behavio*] or [zebrafish (Title) and neurotox* (Title) and behavio*], with the aim of identifying multidisciplinary approaches that also include behavioural analysis.

SUPPLEMENTARY TABLE S2 Main genes related to neurotoxicity analysed in studies conducted on zebrafish. The literature search was conducted with the same criteria described in the caption of Supplementary Table S1. The table shows some reference works for each gene.

SUPPLEMENTARY TABLE S3 Behavioural tests performed on adult zebrafish in the neurotoxicity studies reported in Supplementary Table S1.
